# Maternal Microbiota, Early Life Colonization and Breast Milk Drive Immune Development in the Newborn

**DOI:** 10.3389/fimmu.2021.683022

**Published:** 2021-05-13

**Authors:** Cristina Kalbermatter, Nerea Fernandez Trigo, Sandro Christensen, Stephanie C. Ganal-Vonarburg

**Affiliations:** Universitätsklinik für Viszerale Chirurgie und Medizin, Inselspital, Bern University Hospital, Department for BioMedical Research (DBMR), University of Bern, Bern, Switzerland

**Keywords:** microbiota, innate immune system, pregnancy, gestation, early life, birth, breast milk, neonate

## Abstract

The innate immune system is the oldest protection strategy that is conserved across all organisms. Although having an unspecific action, it is the first and fastest defense mechanism against pathogens. Development of predominantly the adaptive immune system takes place after birth. However, some key components of the innate immune system evolve during the prenatal period of life, which endows the newborn with the ability to mount an immune response against pathogenic invaders directly after birth. Undoubtedly, the crosstalk between maternal immune cells, antibodies, dietary antigens, and microbial metabolites originating from the maternal microbiota are the key players in preparing the neonate’s immunity to the outer world. Birth represents the biggest substantial environmental change in life, where the newborn leaves the protective amniotic sac and is exposed for the first time to a countless variety of microbes. Colonization of all body surfaces commences, including skin, lung, and gastrointestinal tract, leading to the establishment of the commensal microbiota and the maturation of the newborn immune system, and hence lifelong health. Pregnancy, birth, and the consumption of breast milk shape the immune development in coordination with maternal and newborn microbiota. Discrepancies in these fine-tuned microbiota interactions during each developmental stage can have long-term effects on disease susceptibility, such as metabolic syndrome, childhood asthma, or autoimmune type 1 diabetes. In this review, we will give an overview of the recent studies by discussing the multifaceted emergence of the newborn innate immune development in line with the importance of maternal and early life microbiota exposure and breast milk intake.

## Introduction

Our understanding of how microbial communities can influence health and disease of their host has significantly improved in the last decades. This boost of scientific discoveries in the microbiome field has been facilitated mainly by the availability and affordability of different techniques, such as gnotobiology, next-generation sequencing (NGS), and metatranscriptomics. Evidence linking the microbiome with the pathophysiology of diseases, for instance, such as inflammatory bowel diseases (IBD) (1–3), cancer (4, 5), obesity (6, 7), type 2 diabetes (8), and neurological disorders (9, 10) is increasing and future research in the field will in-depth dissect how the microbiota and changes in its composition lead to multiple effects in the host.

**Figure 1:**
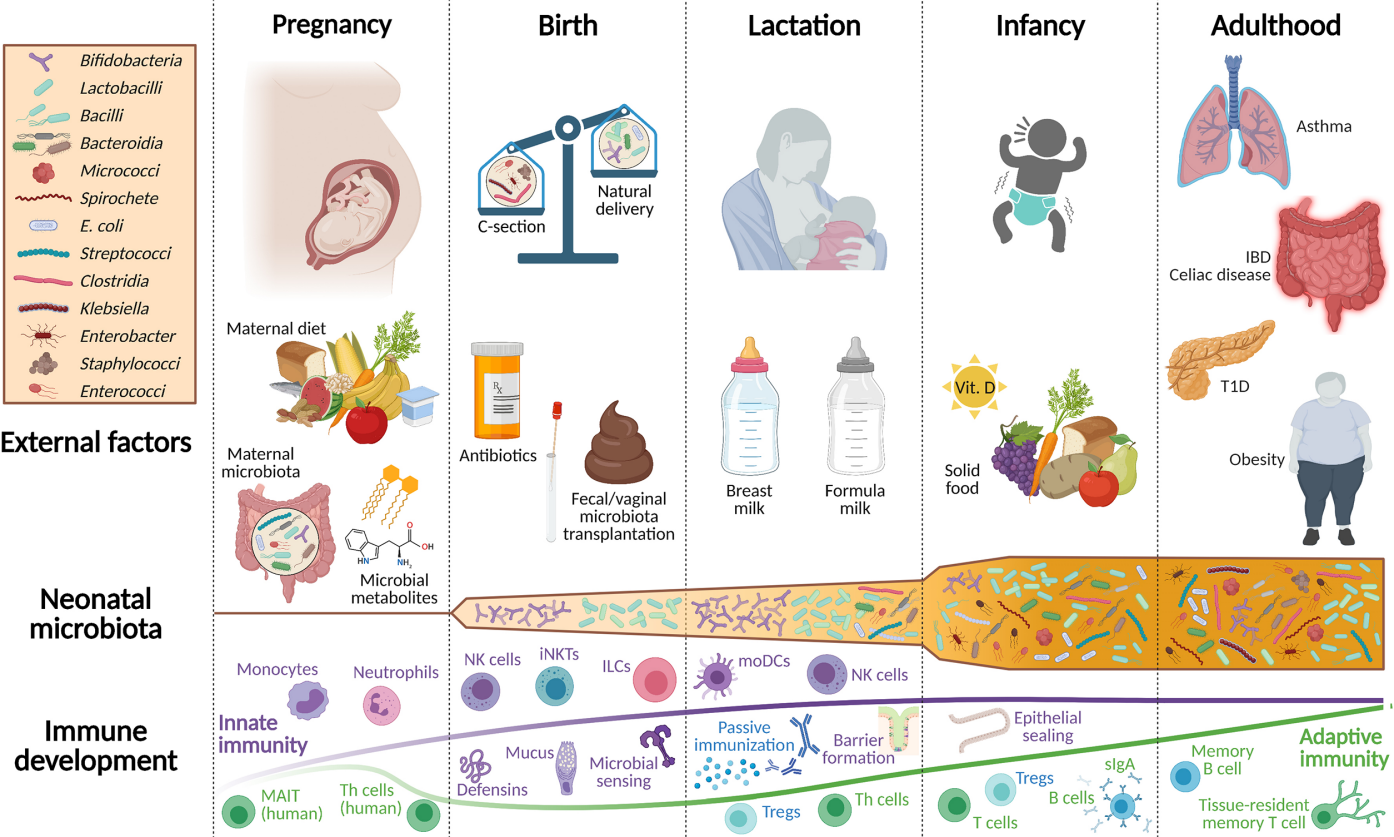
Overview of environmental factors shaping the development of the newborn microbiota and mucosal immune system. Throughout pregnancy, fetal immune development is supported by microbial metabolites originating from the maternal microbiota and by dietary compounds. Innate immune cell populations, such as monocytes, ILCs, and neutrophils belong to the most affected immune cells at this stage. Only at birth, the emerging immune system of the newborn is confronted with live bacteria and thus, is still dependent on maternal protection, which is ensured through breastfeeding. Apart from passive immunization *via* breast milk, neonatal iNKTs, NK cells, ILCs, and the gastrointestinal epithelial barrier protect against invading pathogens and promote the beneficial interplay with the neonatal microbiota. The adaptive immune system in mice develops primarily postnatally, while a component of adaptive immunity is already present in the human fetus. Birth mode, feeding, and the intake of antibiotics are additional factors that shape the early life microbiota and the neonatal immune system. During the subsequent weaning reaction, when solid food is introduced to the infant’s diet, a tremendous shift occurs in its intestinal bacterial community composition. Consequently, the microbiota is no longer represented by Bifidobacteria and Lactobacilli, but increases in metabolomic diversity evolving to a more adult-like microbiota that is established during this early period of life. This period is often also called the window of opportunity since particularly during this time, external cues have a profound impact on life-long health, the evolving microbiota, and the mucosal immune system. IBD, inflammatory bowel disease; sIgA, secretory immunoglobulin A; ILCs, innate lymphoid cells; iNKTs, invariant natural killer T cells; MAIT, mucosal-associated invariant T cells; moDCs, monocyte-derived dendritic cells; NK, natural killer cells; Th cells, T helper cells; Treg, regulatory T cells; T1D, type 1 diabetes.

Birth marks the start of colonization with microbial communities. Many factors are known to influence the microbiota composition in these early days, e.g. birth mode (11), antibiotic treatment during pregnancy (12, 13), or infancy (14, 15), maternal diet (16), breast- or formula feeding (17, 18), and the introduction to solid food (19), while the host genetic background is estimated to only shape about 9% of the intestinal microbiota (20, 21). The microbiome of an infant in its first three years of life is clearly distinguishable from an adult microbiome by a lower diversity index reflected in half as many operational taxonomic units (OTUs) compared to adults and higher interindividual variability (22, 23). Dominant bacterial taxa in the first weeks of life of a newborn include *Enterococcacae*, *Clostridiaceae*, *Lactobacillaceae*, *Bifidobacteriaceae*, and *Streptococcaceae*. In the first months of life, *Bifidobacteriaceae* thrive since they feed on oligosaccharides, which are highly abundant in maternal milk, the main energy source of newborn babies. During weaning, when solid food is introduced, the abundance of *Bifidobacteriaceae* decreases, while *Bacteroides*, *Ruminococcus*, and *Clostridium* become more prevalent (**Figure 1**) (24).

The enormous impact of the microbiota on the development of the immune system was in the spotlight early on. Pioneers like René Dubos, Russell Schaedler, and Dwayne Savage have revealed the importance of the gastrointestinal microbiota and its interaction with the host immune system (25–28). Here, we want to guide through the manifold changes that occur in the microbiota during early life and how this leads to a temporally layered postnatal establishment of the intestinal immune system, starting from gestation, *via* birth, followed by lactation, and the first years of life (**Figure 1**). A strong focus has been put on reviewing literature on innate immune development.

## The Innate Immune System in the Gastrointestinal Tract

The first line of defense against invaders in the gastrointestinal tract includes the mucus layer, the intestinal epithelial cell layer, and hematopoietic immune cells, either scattered throughout the lamina propria or settled as intraepithelial lymphocytes, all of which extensively interact with the microbiota as well as with microbial and diet-derived metabolites.

**Figure 2:**
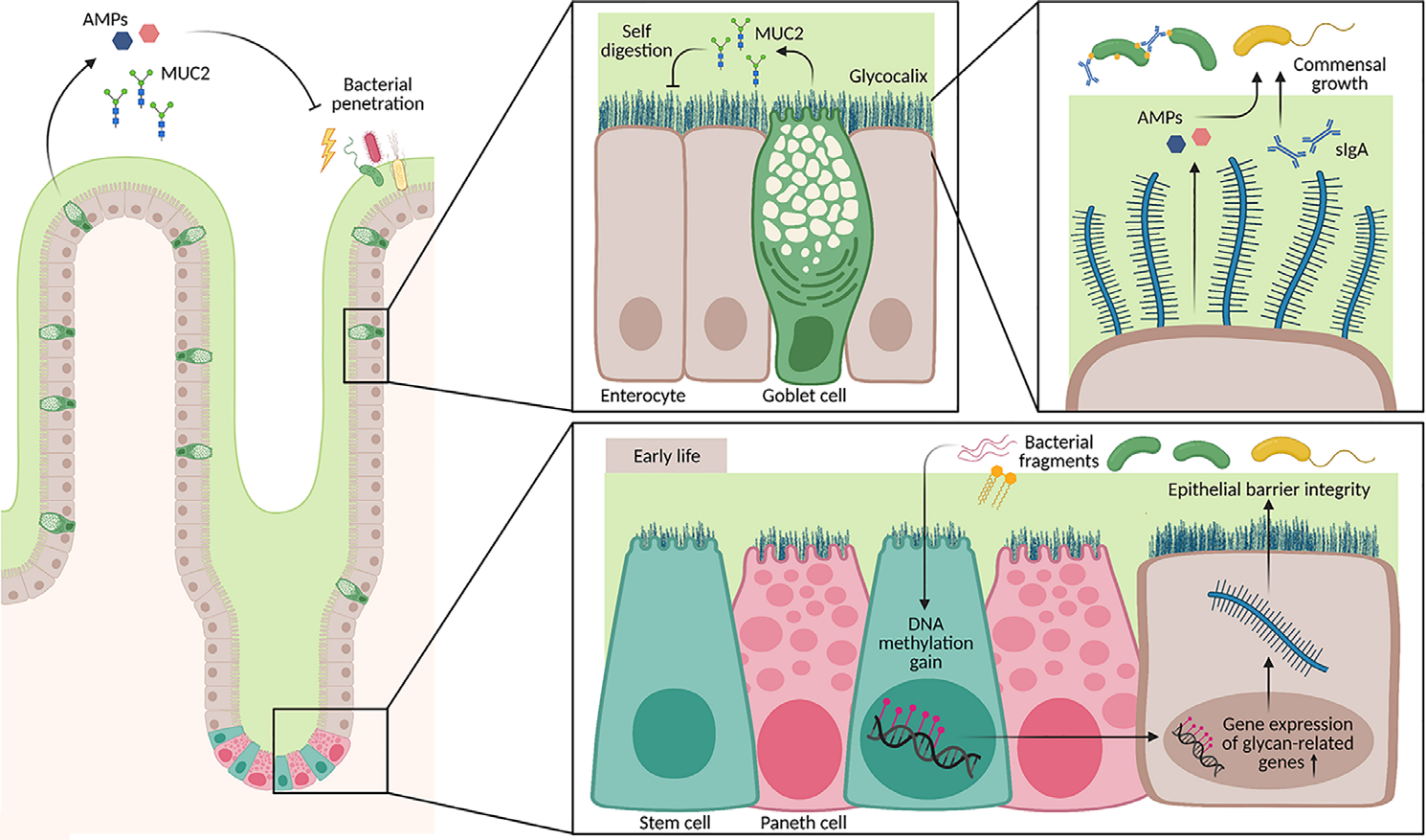
Architecture of the mucus layer in the small intestine. The small intestine has a loose mucus layer (illustrated in green), which keeps commensals at distance. In addition, the epithelium is covered by a glycocalyx, a dense layer composed of secreted mucus proteins (MUC2) that is attached to epithelial cells *via* the transmembrane part. These layers do not only protect from bacterial penetration, but also from self-digestion of host intestinal tissue. Secretion of AMPs by Paneth cells and other epithelial cells as well as sIgA by plasma cells regulate the growth of different commensal bacterial strains. Furthermore, signals from the neonatal microbiota shape the DNA methylome in intestinal stem cells from birth until weaning. Genes associated with cell glycosylation are particularly affected by a DNA methylation gain, which also correlates with an increase in gene expression. Hence, barrier integrity during early life is additionally ensured through epigenetic remodeling triggered by the microbiota.

Mucins build a physical wall that separates the host tissue from the microbial community in the lumen. The large, highly O-glycosylated mucin proteins are either covering the apical surface of enterocytes or secreted into the lumen by goblet cells as gel-forming mucus (**Figure 2**). The small intestine has a single unattached mucus layer, whereas in the colon and the stomach, two layers exist. There, the inner layer is attached to the epithelium, while the outer layer is unattached and less dense. The mucus fulfills different important functions: (1) It protects against the invasion of pathogens, (2) builds a physical barrier between microbial consortia and the host tissue, (3) protects against self-digestion (for example in the highly acidic environment of the stomach), and (4) directly modulates the expansion of different bacterial strains and the composition of the whole community (**Figure 2**). Mucins do not only protect from too close contact to the commensal consortia but also serve as a nutrient source for some bacteria that possess glycosidases; These glycosidases enable bacteria to cleave mucin 2 (MUC2) proteins and use them as an energy source. In turn, bacteria release short-chain fatty acids (SCFAs) that are beneficial for the host (29–31). The mucus is bathed with defensins, different antimicrobial peptides (AMPs) originating from Paneth cells, and secretory immunoglobulin type A (sIgA) deriving from plasma cells in the lamina propria. On the other hand, the microbiota affects mucus property and function (32, 33).

**Figure 3:**
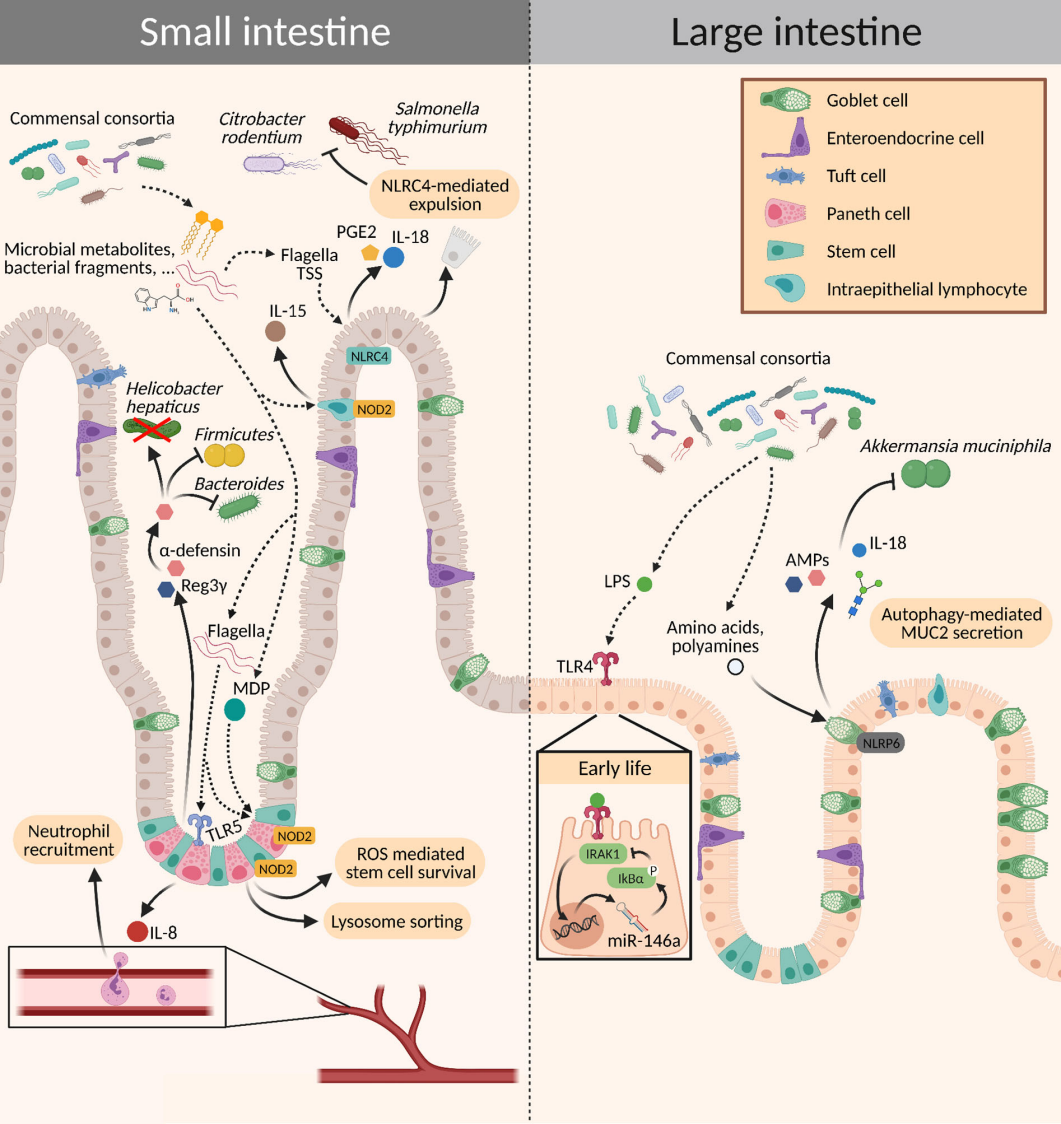
Crosstalk between the microbiota and intestinal epithelial cells. Different signaling molecules from the microbiota bind to PRRs expressed on the epithelium, which subsequently activate innate immune mechanisms. This activation can elicit a wide range of effects: It may trigger a pro-inflammatory state for the elimination of pathogens or induce tolerance to commensals by increasing the production of mucins and AMPs, promoting epithelial cell turnover, and mediating stem cell survival. AMPs, antimicrobial peptides; MDP, muramyl dipeptide; PGE2, prostaglandin E2; ROS, reactive oxygen species; TSS, type secretion system.

The mucosal immune system further comprises the single epithelial cell layer, the underlying lamina propria, and the organized lymphoid structures, such as Peyer’s Patches, isolated lymphoid follicles, and mesenteric lymph nodes (MLNs). Different hematopoietic cells of the innate immune system can be found in the mucosa including mononuclear phagocytes, intestinal macrophages, intestinal dendritic cells (DCs), eosinophils, mast cells, and innate lymphoid cells (ILCs) (**Figure 1**). They sense the presence of microbes through pattern recognition receptors (PRRs) both in the context of an infection with a pathogen and under homeostatic conditions. Many such PRRs and their signaling pathways have been identified in the last decades, including Toll-like receptors (TLRs), C-type lectins (CTLs), nucleotide-binding oligomerization (NOD)-like receptors (NLRs), RIG-I-like receptors (RLRs), and others. These PRRs bind to pathogen-associated molecular patterns (PAMPs) for example lipopolysaccharides (LPS), an outer cell wall component of gram-negative bacteria (**Figure 3**).

## The “Window of Opportunity”

First hints of the possible existence of a “window of opportunity” in early life during which environmental influences can have long-lasting effects on microbiota composition, immune regulation, and disease susceptibility of the host, came from epidemiological studies. Scientists observed a positive correlation between higher hygiene standards in industrialized countries and a rising incidence of autoimmune and allergic diseases (34, 35), which was followed by studies that suggested a reduced exposure to a microbial-rich environment as a possible cause. For example, growing up on a farm, being representative for a microbial-rich environment, prevents the development of allergic asthma (36–39) and this effect has been shown to be long-lasting (40). Furthermore, optimal nutrition is pivotal for both the pregnant mother and her unborn child, as perturbations in this critical time pose the fetus at risk of later developing a plethora of chronic disorders, such as metabolic syndrome, type 2 diabetes, coronary heart disease, adiposity, and osteoporosis (41–44). Until now, therapeutic interventions – for example *via* fecal transplantation – were of limited success, one reason why much effort is taken to further dissect the causal mechanisms of how such early events can lead to later disease onset. The microbiota certainly is a key player during this window of opportunity. Early colonization during this critical time window by a microbial consortium is crucial for the proper development of the immune system and has been demonstrated in various mouse models (14, 45–48). It seems that the window of opportunity in mice closes around the time of weaning. In humans, we know that a stable microbiota composition is established at the age of 2-3 years. However, no clear data are yet available to assess the exact time when the window of opportunity closes. While the immune system of the child predominantly matures in the first few years after birth, it is still strongly shaped later during childhood. Due to the distinct needs at different stages in life, the structural organization of various immune layers at the intestine has been identified. In neonatal mice, there is no established crypt-villous axis, and their epithelium is characterized by a lower turnover compared to adult mice (48–50). Additionally, there are almost no mature Paneth cells present in neonatal mice and the mucus layer is much thinner – it is only around the time of weaning when a reliable mucus shield against invaders is established (48). Further, PRRs are expressed in an age-dependent manner. For example, TLR4 expression is increased in the prenatal period and decreases at term, whereas TLR9 expression is reduced during gestation and increases after birth. This pattern of expression for TLR4 and TLR9 is inversed in tissue from infants suffering from necrotizing enterocolitis (NEC) and might be of pathophysiological relevance (51). TLR3 expression is low at birth and increases during the postnatal period (52). Sensing of LPS in the neonatal intestine *via* TLR4 leads to expression of the microRNA miR-146a which maintains phosphorylation of IκBα, subsequently inhibiting IRAK expression and resulting in LPS tolerance (53, 54) (**Figure 3**). One could hypothesize that age-dependent expression of PRRs may represent a strategy of the host to push colonization of the mucosal sites towards beneficial commensals and it is possible that altering expression levels of different PRRs in a timely manner helps to fine-tune age-dependent requirements of the host, e.g., in terms of nutrients provided by certain bacteria.

What has started as epidemiological correlation-based observations has developed into in-depth experimental research. These scientific investigations have revealed mechanistic insights into how events during this critical window affect long-term health of the host. The window of opportunity not only opens after birth but already during pregnancy, having manifold effects on the developing fetus, in ways that we will discuss next.

## Gestational Imprinting of the Neonatal Intestinal Innate Immune System

Adaptations of the female body during pregnancy are remarkable, affecting all organ systems and including the development of the placenta, a highly specialized organ that provides an anatomical separation of the fetus and the mother for preventing immunogenicity of the mother against the fetus and vice versa. At the same time, this complex organ ensures the maternofetal exchange of molecules, including those originating from the maternal microbiota (55). In recent years, much attention has been drawn to the possible existence of a placental microbiome itself.

### Is There a Placental Microbiome?

Whether the placenta harbors a microbial community is a matter of debate and has started when a study from the Versalovic group in 2014 has challenged the paradigm of a sterile womb. They performed 16S sequencing on human placental samples and detected a microbial community (56). Before that, others have found bacteria in the human placenta during term (57, 58) and preterm deliveries (58). Bacteria have also been identified from human umbilical cord blood (59), meconium (60), and amniotic fluid (57). Additionally, genetically labeled *E. faecium* was administered orally to pregnant mice and subsequently isolated from the culture of amniotic fluid (59) and meconium (60), many studies followed claiming the existence of a placental microbiome (61–64).

Immediately after the Versalovic group published their paper in 2014, Harvey Kliman pointed out that the sole detection of DNA does not provide evidence for the existence of living microbes (65). Over time, it became more and more obvious that contamination issues (66, 67) and the Test-Kit’s own “microbiome”, the so-called “kitome” (68), represent big challenges in the search for a microbiota inhabiting the placenta. Subsequently, several groups had a closer look and performed even more careful evaluations (1) by adding controls at every step of the process, (2) only including samples of cesarian (C)- section-derived tissue to reduce the risk for contamination during the birth process, (3) combining high-throughput sequencing with qPCR and bacterial culture, (4) comparing the bacterial taxa from those found in the close environment (e.g. the processing room), and (5) subtracting the taxa that overlapped with the kitome. Scientists were not able to detect a placental microbiome by including the above-mentioned precautions (68–73). However, the issue does not seem settled, as a recently published paper claims to have detected bacterial DNA and viable bacteria in the fetal intestine by using 16S rRNA gene sequencing, qPCR, electron microscopy, and bacterial culture (74), which re-sparked the controversy whether a placental microbiome exists (74–76).

From a biological perspective, we doubt the presence of an established microbial community in the placenta (55, 77). As elegantly reasoned by Walter & Hornef, «multi-layered contextual evidence» has not been taken into account by proponents of the *in utero* colonization hypothesis, but most studies have focused on sequencing techniques only (78). They further emphasized that there is no overlap between the bacterial taxa detected *in utero* in the different sequencing studies but almost a congruence between the bacterial taxa identified *in utero* and the controls (78).

### Maternofetal Exchange Influencing the Development of the Neonate’s Mucosal Innate Immune System

Even though the existence of a placental microbiota is questionable, the transport of commensal bacteria-derived metabolites *via* the placental barrier is well established. The gut maternal microbiota plays an important role in this maternofetal molecular transfer and is thereby able to modulate fetal development (79). Our group showed that reversible colonization of germ-free females with a genetically modified *E. coli* strain (80) during pregnancy induced distinct changes in the intestinal innate immune system of the offspring (77). This was dependent on the transfer of maternal microbiota-derived aryl hydrocarbon receptor (AhR) ligands (81), which stimulated the proliferation of innate lymphoid cells type 3 (ILC3s) (77). ILC3s are crucial for maintaining the gut epithelial barrier and host defense by the production of IL-22 and IL-17 and subsequently inducing the secretion of AMPs (82, 83). Moreover, the expression of genes involved in epithelial cell differentiation, integrity, and homeostasis was altered in small intestinal epithelial cells of the offspring born to mothers who had experienced reversible colonization during pregnancy (77). Our group recently reviewed how manifold metabolites processed by the maternal microbiota can reach the fetus and affect its development and physiology (55).

Antibiotics and other drugs can indirectly influence the maternal microbiota-fetus crosstalk, by altering the microbiota composition and subsequently the metabolite-pool derived from the microbiota. Perinatal antibiotic exposure reduced Nrp-1^-^RORγt^-^Foxp3^+^ regulatory T cells (Tregs) in the offspring and was irreversible after weaning (84). Another study found lower levels of IL-17 and granulocyte colony-stimulating factor (G-CSF) in the intestine of antibiotic-treated dams. Their neonates exhibited decreased numbers of circulating and bone marrow neutrophils as well as granulocyte/macrophage-restricted progenitor cells in the bone marrow (82). However, the offspring of antibiotic-treated non-obese diabetic dams were protected against the development of type 1 diabetes through mechanisms of alteration in the microbiota composition and induction of tolerogenic antigen-presenting cells (APCs) (85, 86). In humans, a population-based Danish cohort study found a positive correlation between antibiotic exposure during pregnancy and a risk for severe infections in children (87). Antibiotic treatment during pregnancy was further associated with an increased risk for very early onset of IBD in the offspring (88).

An important side note is the observation that a healthy pregnancy leads to changes in the microbiota composition that resembles a dysbiotic composition – however, in the context of pregnancy with its unique needs and requirements, these adaptations are physiological (89, 90). For example, *Faecalibacterium*, which is a SCFA producer, decreases in abundance in the last trimester of pregnancy. This decline of *Faecalibacterium* has also been observed in populations with metabolic syndrome (91). Overall, pregnancy is associated with a decrease in microbial diversity and richness, and an increase in bacterial load with an expansion in *Proteobacteria* and *Actinobacteria* (92, 93). This shift in the microbiota of pregnant women is subjected to adjustments in dietary habits, which are accompanied by changes in the pool of bacterial metabolites to fully support the development of the fetal immune system. A fiber-rich diet during pregnancy protected the offspring against the onset of asthma, probably *via* inhibition of histone deacetylase 9 (HDAC9) mediated by acetate resulting in higher gene transcription of *Foxp3* in Tregs. They further lowered frequencies of eosinophils and macrophages in the blood and bronchoalveolar lavage fluid as well as serum IgE levels of the offspring (94).

1,25-dihydroxyvitamin D_3_ plays an important role in epithelial barrier integrity. Mice with vitamin D deficiency and *C. rodentium* challenge demonstrated increased colonic hyperplasia and epithelial barrier dysfunction (95). Malnourished pregnant mothers, specifically in 1,25-dihydroxyvitamin D_3_, might be at higher risk for developing intestinal infections, which poses a substantial risk to the unborn child. Another study found that lymphocytes isolated from the cord blood downregulated *TLR1*, -*2*, -*4*, -*6*, and -*9* upon supplementation with high doses of 1,25-dihydroxyvitamin D_3_ during pregnancy (96). In mice, maternal dietary-derived retinoic acid, the active form of vitamin A, influences secondary lymphoid development in the offspring as lymphoid tissue inducer (LTi) cells, a subset of ILC3s (97). Mechanistic insights on a molecular level for the relation between prenatal nutrition and intrauterine immune development come from a study investigating metastable alleles in a Gambian human population with seasonal variations in food supply. A metastable allele, VTRNA2-1, was differentially methylated between offspring from mothers, which were either at conception when food was available in adequate or insufficient amounts. Strikingly, VTRNA2-1 has been identified to play a role in viral immunity (98), which reflects common observations linking undernutrition with higher infection rates.

While the mentioned examples illustrate that the maternal microbiota can affect the development of the offspring’s immune system already *in utero*, additional studies are needed in the future to better understand this crosstalk.

## The Interplay of Early Life Colonization and Neonatal Immunity

When it comes to the development and maturation of the newborn’s immune system to guarantee lifelong health, the immediate period after birth is as important as the gestational stage. Although several key steps regarding innate immune development take place already *in utero*, many others require postnatal antigen exposure to evolve. NK cells and ILCs are present already at birth and subsequently expand and even reach higher frequencies than in adulthood. This ensures that the newborn is prepared against immediate threads and protected against infections early in life (99–103). Even though the neonate’s innate immune system is capable to mount an immediate response against potential pathogens right after birth, it still has to mature in coordination with the microbiota and many other environmental factors (48, 104–106). In contrast, the murine adaptive immune system develops predominately postnatally. This is not completely transferrable to the human organism. Data are available that demonstrate the presence of adaptive immune cells in the developing fetus. Teichmann and colleagues detected thymic T cells in the fetus as early as 7 weeks post conception (107), while effector CD4^+^ T cells were described in the intestine of the developing fetus during the second trimester of pregnancy (108). In addition, mucosal-associated invariant T (MAIT) cells are already present during *in utero* development of humans and can protect the newborn baby from infections (109).

### Are the Newborn Microbiota Development and Immune Maturation Shaped by the Birth Mode With Long-Term Consequences on Human Health?

The event of birth represents the change from the sterile environment *in utero* to the rapid colonization of all body surfaces. For a vaginally born baby, this is initiated by vertical transmission of microbes when passing the birth canal and primarily includes microbes inhabiting the maternal gut lumen (110). A thorough metagenomic shotgun sequencing analysis of fecal samples collected at different timepoints from full-term infants during their first year of life points out the dynamics and importance of the microbiota early in life. As previously mentioned, the complexity of the gut microbiota increases during the first year after birth (**Figure 1**). Simultaneously, the composition of the gut microbiota progressively resembles the maternal gut microbiota and ultimately develops into the adult gut microbiota (13, 23, 111).

This process of early life colonization can be perturbed by external factors, for example, when a baby is delivered by C-section. Babies born *via* surgical delivery share around 30% less bacterial species with their mother than naturally born babies, indicating different sources of gut colonizers. Indeed, infants born *via* C-section harbor an increased number of species usually colonizing the skin (*Staph. saprophyticus*) or circulating in the hospital (*Enterococcus faecalis*, *Enterobacter cloacae*, *Klebsiella pneumoniae*, and *Clostridium perfringens*) (13, 112). Most often, acquired from the hospital environment, these strains are opportunistic pathogens, also relevant in nosocomial infections and harboring antimicrobial resistance genes (112). At the phylum level, the microbiota of babies born *via* C-section is dominated by *Firmicutes* and *Proteobacteria*, with a shift to fewer *Bacteriodetes* and *Actinobacteria* (113). Additionally to the missing passage through the birth canal, also the use of antibiotics, which is the first-line pharmacological therapy intrapartum, disturbs the microbial colonization of the neonate at birth. Within the first year of life, the microbiota of C-section babies is able to recover but may also persist for longer (13, 111, 113–116). Therefore, it is still highly debated whether surgical delivery has life-long consequences. A comprehensive study performed in Denmark associated a multitude of inflammatory diseases to C-section delivery. Over 2.5 million candidates were followed from birth up to 40 years of age. Indeed, participants born *via* C-section were at a higher risk to develop diabetes, arthritis, celiac disease, or IBD. Nonetheless, a correlation between a distinct microbiota early in life as a consequence of cesarean section and the onset of immune-mediated etiologies later in life could not be disentangled (117). In a recently published study, Stockholm and colleagues addressed this relationship (110). They could demonstrate that infants with a long-term C-section-associated microbiota composition suffer from a higher susceptibility to childhood asthma or an increased risk of allergic sensitization marked by high IgE. Children who retained a C-section microbiota profile at the age of one year also mounted a different immune response during episodes of acute airway symptoms, determined by lower levels of immune mediators, such as TNF-α, IL-4, IL-13, or IL-1β. It is not yet clear, which changes specifically in the gut microbiota are the key factors driving these allergic phenotypes (113). In contrast, children born *via* C-section, who could normalize their microbial colonization pattern during their first year of life, were not affected by either a higher risk for childhood asthma or allergic sensitization early in life. Hence, other impacts on gut microbial composition, such as the contact with the maternal microbiota from body sites other than the gut or having older siblings in the family might play a pivotal role in maturing the neonatal microbiota towards a less sensitizing composition (110, 113, 118, 119).

Since the immune system is largely evolving during this window of opportunity, a C-section-associated composition could interfere with the healthy development of the immune system and conclusively explain the threefold risk to develop childhood asthma or other immune-related disorders after cesarean delivery (113, 117). Specifically, the development of the regulatory immune system is affected by the birth mode, as shown in several murine studies. Mice born *via* C-section had stunted levels of Tregs in the spleen and MLNs and reduced systemic IL-10 levels until adulthood (120, 121). On the other hand, the proportion of invariant natural killer T cells (iNKT) was increased in the colon, as well as their expression of *Il4* and *Il15*. A prebiotic diet was able to reestablish reduced numbers of iNKTs similarly to vaginally delivered mice. However, the levels of Tregs were unaffected and remained low (121). The causative effect of high iNKTs and the early life microbial colonization pattern after C-section in humans has yet to be elucidated. Notably, normal iNKT cell levels in the colonic lamina propria could only be restored when germ-free mice were colonized in the first few weeks of life but not at later time points (46). Additionally, germ-free mice and mice delivered *via* C-section were more susceptible to iNKT cell-mediated oxazolone-induced colitis, a murine model for IBD (46, 122).

The practice of C-section, specifically without any urgent medical indication, is increasing worldwide, and in wealthy countries even with a skyrocketing prevalence (123). Hence, it is also of interest to overcome the imbalanced mother-to-neonate transmission after surgical delivery. In a proof-of-concept safety study, Korpela and colleagues orally transplanted fecal microbiota, collected from a total of 7 mothers, to their C-section-born neonates. Fecal microbiota transplantation (FMT) could shift the gut microbiota towards a vaginally born colonization pattern, including the restoration of a healthy *Bacteroides* level (124). Additionally, FMT alleviated the abundance of opportunistic pathogens characteristic of a C-section microbiota (112, 124). Even though the proof-of-concept safety study displayed benefits for FMT after cesarean delivery, such practices should be regarded with caution. The mother could harbor potential pathogens or viruses, with which her immune system is able to cope, but not the immature immune system of the newborn. Therefore, thorough microbiota profiling beforehand is crucial. In the mentioned study, for example, only 7 out of 17 mothers were selected after careful examination (124). A different approach for microbiota restoration in newborns is vaginal microbiota transfer at birth. Nevertheless, the major source for neonatal gut colonizers is the maternal intestinal microbiota, hence, not surprisingly vaginal swabbing at birth was unable to durably establish a microbiota similar to vaginally born babies (110, 124). These findings stand in contrast with the conclusions drawn by Dominguez and colleagues. Exposure of newborns with vaginal fluids at birth, exhibited a vaginal microbiome-like signature during the first week of life, which was similar to vaginally born babies and to the vaginal microbiota of the mother (125). Hence, even though the effects of C-section on the development of the newborn microbiota and its immune maturation are well established, the options to adjust the imbalance during the window of opportunity and particularly its consequences on long-term microbiota composition and human health are still a matter of debate and extensive research.

### The Effect of Environmental Cues, Such as Xenobiotics, Vitamins, or Other Dietary Agents on Neonatal Immune Development and Susceptibility to Immune-Mediated Diseases

#### Antibiotic Treatment

The postnatal maturation of the immune system is highly sensitive to environmental factors. Preterm babies are particularly weak and complications at this stage are still a major cause of neonatal death (126). Preterm babies often need to undergo empirical antibiotic treatment in the neonatal intensive care unit (NICU), leading to nosocomial late-onset sepsis (LOS), which can appear 3 days after birth or later (127, 128). Empirical antibiotic treatments include generally broad-spectrum antibiotics, including Vancomycin and thus, the increasing prevalence of antimicrobial-resistant pathogens, such as the multidrug-resistant *Staphylococcus capitis* clone (NRCS-A) is alarming. Not only because NRCS-A is exceptionally disseminating in NICUs, but also due to its probable relevance in LOS pathogenesis (129, 130). Approaches, such as the MinION seq platform, aim to rapidly (in less than 5 h) identify the pathogenic species and their antimicrobial-resistant genes to reduce broad-spectrum antibiotic treatment early in life and specifically target LOS in the newborn patient (131). Neonatal antibiotic treatment impedes the building microbiota and may set the basis for pathological colonization and finally LOS. Likewise, the maturation of the immune system is impaired due to its indispensable relationship with the microbiota. In a murine model, Niu et al. could unravel the effects of antibiotic use and LOS on the innate immune system (83). Specific pathogen-free (SPF) dams were treated with antibiotics either shortly (3 d) or for a prolonged period (7 d) while they were nursing their neonates, resulting in antibiotic exposure of the neonates through their mother. This experimental design mimics the situation of preterm babies receiving empirical antibiotic treatments resulting in a stunted microbiota. Both, in short, and in prolonged antibiotic exposure and similar to human babies born *via* C-section, *Proteobacteria* expanded persistently, whereas *Enterobacter* and *Enterococcus* species could only translocate to the spleen and the liver after long-term antibiotic exposure. Due to this shift in neonatal microbiota, *K. pneumoniae* was able to colonize the neonatal gut and additionally translocate systemically, resulting in sepsis. Reduced bacterial signals diminished epithelial TLR2 and TLR4 gene expression, which commonly drive ILC3 expansion. Ultimately, long-term antibiotics also diminished the ILC3 population in the lamina propria. Since microbiota restoration reestablished ILC3s and rescued the antibiotic-treated pups from sepsis, this innate immune population contributes to the important protection of the neonate after birth. However, changes in the abundance of ILC3s through neonatal antibiotic exposure by treating the dams might also be explained by changes in the maternal microbiota or breast milk itself (83). The importance of maternal microbiota-induced intestinal ILC3s in regulating early life colonization was also demonstrated by our group as discussed earlier (77).

#### Early Life Vitamin D Supplementation

Vitamin D deficiency during the first years after birth was previously associated with an increased risk to develop asthma, eczema, or atopic sensitization during childhood (132, 133). One of the determinants of risk for childhood asthma development might be early nasopharyngeal colonization with pathogenic *Streptococcus* species (132). The immunological mechanism behind, most probably involves the development of tolerogenic DCs and Tregs after repeated exposure to aeroallergens, which affects normal maturation of pulmonary function, followed by cumulative airway tissue damage (134, 135). Under healthy conditions, CD11b^+^ migratory DCs are sensitized after house dust mite and microbial LPS exposure, which prompts them to dampen type 2 T helper (Th2) cell differentiation through TNF-α signaling. This process is well-developed in adult mice, in contrast to younger mice, where the threshold for sensitization is decreased. Thus, young mice are more prone to develop asthmatic inflammation due to the weakened response of DCs towards microbial low-dose LPS stimuli, which then insufficiently suppress Th2 cell development (136). Latest studies report altered differentiation and activation of several T cell subsets, including Th1 and Th2 cells after oral vitamin D supplementation or UV exposure in neonates. Especially the differentiation of naïve CD4^+^ T cells into Th2 cells was reduced by lower IL-2 production (137, 138).

Apart from vitamin D deficiency, childhood asthma has also been associated with respiratory viral infections early in life. Therefore, vitamin D supplementation could alleviate asthma risk by reducing viral infections, explained through distinct neonatal IFN-γ production and abnormal neutrophil responses (139, 140). A study performed in Vietnam confirmed that an 8-month supplementation of vitamin D during infancy diminished the frequency of respiratory infections caused by non-influenza viruses, such as rhinoviruses. However, the influenza infection rate remained unaffected by vitamin D administration (141). Collectively, neonatal vitamin D is crucial for the healthy development of the respiratory immune system and notably to improve the communication between respiratory microbial signals, the innate and the adaptive immune system with life-long consequences on asthma susceptibility (133, 136, 137).

#### Short-Chain Fatty Acids and Extensive Gluten Intake During Infancy

Gut commensals play a key role in processing indigestible food components to provide essential vitamins and SCFAs to the host. The period when solid food is gradually introduced coincides with a burst in microbial changes and immune regulatory processes, also known as the weaning reaction. In case of an absent weaning reaction, pathological imprinting occurs, having life-long consequences on host allergy and cancer susceptibility (19). The weaning reaction is dependent on the microbiota and additionally on a specific time window since the reconstitution of germ-free mice at later stages could not rescue from pathological imprinting in the small intestine (19). During a preweaning interval, goblet cell-associated antigen passages (GAPs) are formed and specifically deliver antigens from epithelium-adhering bacteria to the colonic lamina propria. These encounters prime Foxp3^+^ Tregs to protect the neonate from dextran sulfate sodium (DSS)-induced colitis later in life. Essentially, blocking GAPs before weaning inhibited Treg development in the colonic lamina propria and resulted in an impaired establishment of early life bacterial-induced tolerance (142). The microbiota produces SCFAs from dietary fibers, which promote the expansion of RORγt^+^ Tregs in the small intestine and thereby prevent pathological imprinting. Nevertheless, SCFAs alone were not able to induce Tregs in germ-free mice, nor was exclusive bacterial colonization. Hence, additional signals are required and we will display later in this review that the weaning reaction is not solely happening because of the digestion of dietary fibers by gut microbes, but that it depends further on components in the breast milk, which delay the weaning reaction (19).

Dietary fibers are found in plant-derived food. Apart from vegetables and fruits, also barley, rye, and wheat are vital fiber sources. These different grain varieties contain gluten, an important food antigen, which can cause celiac disease in genetically predisposed individuals. Since not all individuals with the celiac-relevant HLA antigen genotype develop celiac disease, environmental factors seem to play an essential role. One currently discussed environmental aspect is the effect of early life gluten intake because the disease often manifests during infancy (143–145). Several studies highlight the association of extensive gluten intake during childhood with an increased risk of developing celiac disease if genetically predisposed (146–148). It is hypothesized that shifts in the microbiota composition during the first year of life precede the disease pathology. These include high abundance of *Bifidobacterium breve* and *Enterococcus* spp., whereas increases in *Firmicutes* and *Bifidobacterium longum* are correlated with a reduced risk. Even though the overall changes in the commensal community were minute, children who later developed celiac disease exhibited a premature microbial diversity and complexity, followed by increased IL-6, reduced sIgA, and TNF-α levels in the feces (149). However, the cause for the microbial shift and its consequences on immune regulation and response to the gluten antigen could again be attributed to different cues *in utero* or after birth, such as the feeding practice (150).

Celiac disease onset as a result of early life development summarizes the importance of a balanced interplay between food intake, gut microbial metabolism, and mucosal immune function, which altogether maintain gastrointestinal epithelial barrier integrity, an innate immune mechanism we will highlight in the next section (149–152).

## Gastrointestinal Epithelial Barrier and Gut Commensals Early in Life

The gastrointestinal epithelium acts as a physical barrier to prevent translocation of pathogens, but it also senses microbial antigens to maintain host-microbial mutualism (153). The oral epithelium is a multilayered, stratified epithelium similar to the cutaneous epithelium, whereas the lower parts of the intestine are aligned with a single layer of cells. Therefore, the epithelial establishment and its crosstalk with immune cells and the microbiota evolve to some extent differently in the oral cavity, the small intestine, and the colon (49, 50, 154, 155).

The oral cavity belongs to the earliest microbially exposed body surfaces. Nevertheless, little is known about the development of its mucosal immune system. Only recently, light was shed on the mutual interplay between microbiota development and local immunity. In contrast to the intestine, neutrophils are highly abundant in the neonatal oral mucosa and due to the increased permeability of the oral epithelium before weaning, they play an important role in the first line of defense against the high microbial load acquired at birth. Upon microbial stimulation, γδ T cells produce IL-17 and induce the recruitment of prenatally established neutrophils. At weaning, these γδ T cells diminish and simultaneously the oral epithelial barrier strengthens by reducing its permeability and upregulating saliva production. With the higher levels of salivary AMPs, the microbial load decreases and the oral epithelium finally matures, having a greater turnover and a reduced expression of microbial recognition receptors, including TLR2 and TLR4, and lower expression of antimicrobial defensins. Conclusively, the early defense mechanisms of the innate immune system against the oral microbiota enable the development of a tolerogenic oral epithelium, which during the neonatal phase is most vulnerable (49).

In the lower intestine, where microbial density increases, luminal metabolites change dramatically during weaning. This newly introduced metabolome not only affects the development of the immune system but also its regulation through epithelial cells by strengthening the physical barrier and supporting epithelial maturation to an absorptive phenotype (19, 156). Identical to the oral cavity, the small intestinal and cecal epithelia reduce the transcription of TLRs and AMPs at the suckling-to-weaning transition (156–158). A recent study by Hornef and colleagues found an age-dependent expression of TLR5, where relative expression was 200-fold higher in newborns compared to adult mice. Importantly, this difference in TLR5 expression was specific to the intestinal epithelium and had a lifelong impact on microbial composition in the gut (158). Specifically, stimulation of Reg3γ production mediated by TLR5 controlled counter-selection of flagellated bacteria and thereby modulated the intestinal microbiota until adulthood (**Figure 3**).

Apart from microbial sensing, the microbiota shapes the functionality and performance of the intestinal epithelial stem cell niche during development. Bacterial exposure at weaning affected gene expression of the erythroid differentiation regulator-1 (*Erdr1*), which is important for stem cell proliferation and regeneration after epithelial damage in the neonatal colon (159). However, already before weaning, microbial metabolites influence intestinal epithelial renewal. Small intestinal organoids stimulated with sterile filtered stool supernatant obtained from term-babies exhibited higher proliferation and accelerated maturation than untreated organoids. Stool supernatants from pre-term babies, having a stunted microbiota composition, were unable to stimulate organoid development (160). From this mixture of microbial metabolites produced by a healthy microbiota, it is the ambitious goal of mucosal immunologists to identify substances to use as possible therapeutic agents to modulate the imbalanced mucosal homeostasis in susceptible newborns. To narrow down the myriad of possible metabolites, analyses on less diverse systems are performed. Monocolonization of murine pups with a particular *Bifidobacterium breve* strain (UCC2003) was able to boost epithelial regeneration by supporting the stem cell niche and increasing epithelial differentiation in the small intestine. Additionally, upregulation of integrins, tight-junction molecules, and increased mucus production in pups after *Bifidobacterium breve* treatment supported its role in strengthening the physical intestinal barrier (161).

The turnover rate of the small intestinal epithelium is slower in neonates than in adult mice (49, 50). This might explain the protection of neonatal mice against inflammation-induced cell shedding after LPS administration. Since apoptotic signaling cascades, including TNF-α production, were intact in neonates, it appears that innate immune mechanisms guided through IFN-γ provide the necessary protection against pathological cell shedding. Again, gut commensals were crucial to orchestrate the immune system towards a tolerogenic phenotype with elevated IL-10 levels to protect from increased LPS-induced epithelial cell death (162).

Long-lasting epithelial homeostasis is partially driven by epigenetic remodeling as a response to microbial stimuli early in life. The most intensively studied metabolite with epigenetic relevance is butyrate, a product from bacterial fiber metabolism. Its supportive role on epithelial sealing, which coincides with the implementation of dietary fibers in combination with its inhibitory effect on histone deacetylases, suggests an important role in regulating epithelial maturation to ensure lifelong mucosal homeostasis (156). Nonetheless, epigenetic remodeling on the level of DNA methylation appears to be more central early in life. Already in 2015, Yu et al. demonstrated that a DNA methylation gain in intestinal stem cells from birth until weaning had lifelong consequences on barrier integrity (163). Genes associated with a methylation gain were involved in intestinal maturation and glycosphingolipid biosynthetic processes. Also, the expression of these genes affecting glycosylation was increased, indicating that the DNA methylation gain is affecting regulatory regions and further supporting the role of epigenetic remodeling in early life barrier formation (163). Glycosphingolipids contribute to the epithelial cell barrier integrity by shaping the glycocalyx of intestinal epithelial cells (**Figure 2**). Furthermore, the glycosylation pattern of intestinal epithelial cells has been directly associated with beneficial effects on age susceptibility to pathogenic bacterial infections, IBD, and cancer metastasis (164, 165). The DNA methylation gain is microbiota-dependent since mice raised germ-free showed a dysregulated DNA methylation development in the colon from birth until adulthood when compared to conventionally raised mice (CNV). Conventionalizing germ-free pups with FMT at postnatal day 25 partially restored the gain in DNA methylation in adulthood (163). The impact of the microbiota on the DNA methylome in colonic or small intestinal epithelial cells is most probably restricted to specific gene sets and does not affect the DNA methylation pattern on a global scale (157, 163). Enhancer elements known to be lowly methylated, so-called low-methylated regions (LMRs) are strongly affected by the microbiota. This DNA methylation analysis stays in agreement with the transcriptomic analysis and indicates that gut colonization integrates DNA demethylation in LMRs early in life with consequences on gene transcription, which finally drives intestinal development and homeostasis. The epigenomic differences between germ-free and CNV mice had functional consequences on gastrointestinal health, confirmed by increased chromatin accessibility after induction of DSS colitis in genes playing a role in the inflammatory response of the colonic epithelium in CNV mice (166). These findings indicate a profound effect of the microbiota on intestinal epithelial cell homeostasis through epigenetic regulation early in life, certainly, an area that requires further analytic investigations in the future (157, 163, 166).

## The Importance of Breast Milk for the Development of the Neonate

**Figure 4:**
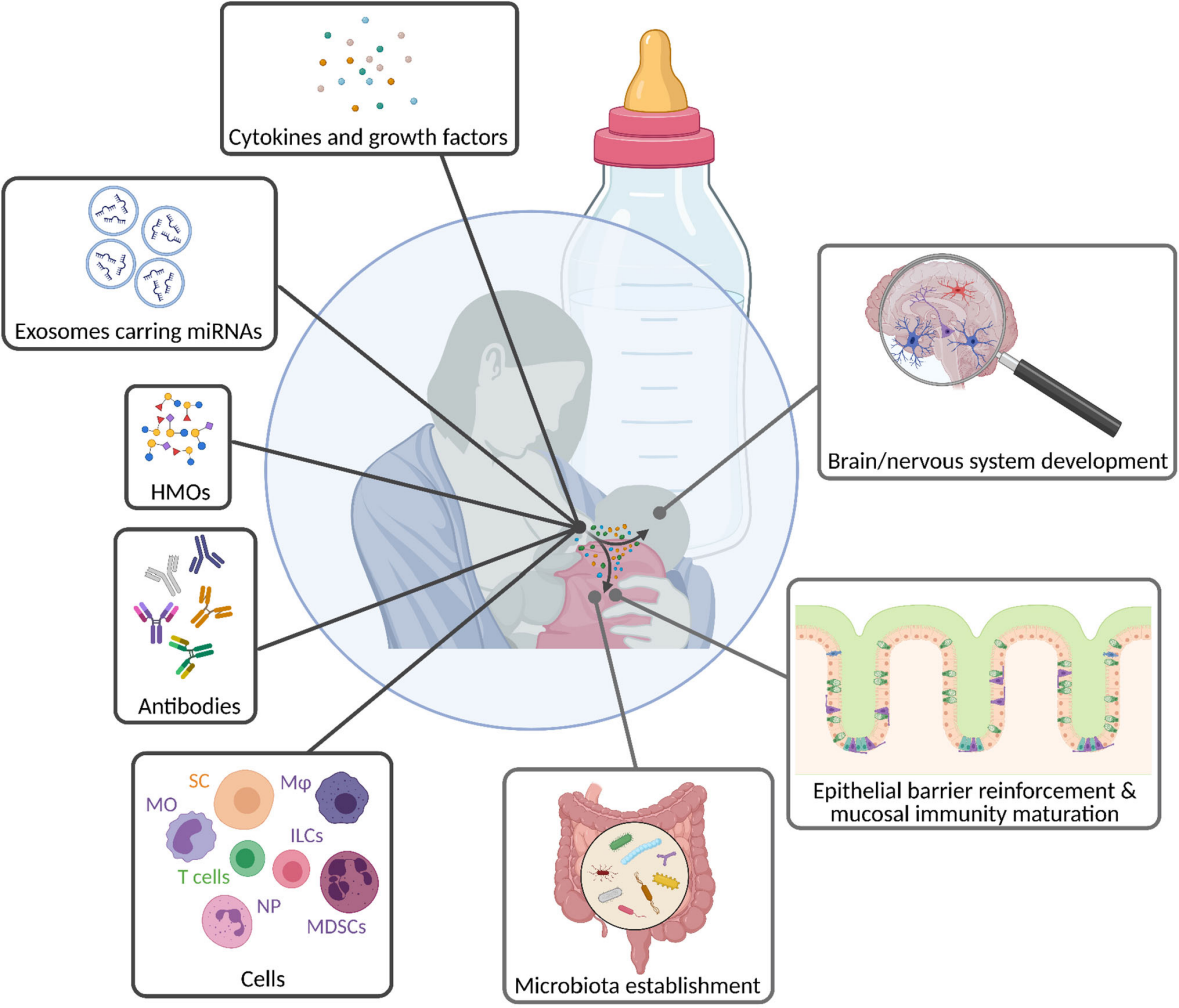
Breastfeeding mediates the transfer of biologically active molecules from the mother to the infant. Breast milk contains a huge diversity of components, ranging from simple sugars, antibodies, and cells to molecules that directly trigger reactions in target cells and/or tissues, such as cytokines, growth factors, and exosomes. Thereby, milk molecules ensure the infant’s well-being by driving innate and adaptive immune maturation, and further contributing to the development of its mucosal and nervous system. HMOs, human milk oligosaccharides; ILCs, innate lymphoid cells; Mφ, macrophages; MDSCs, mononuclear-derived suppressor cells; MO, monocytes; NP, neutrophils; SC, stem cells.

Breastfeeding is one of the most meaningful exposures during early life. It is considered the best nutrition source for the infant as it contains the perfect balance of lipids, proteins, and carbohydrates, as well as high amounts of micronutrients that are crucial for neonatal growth (167–169). Many of the breast milk components are not only a nutrient source but are additionally biologically active, protect the neonate against pathogen- and immune-mediated diseases (**Figure 4**) (168, 170–174), and drive the maturation of its immune system (175, 176). Recently, a human study showed that breast milk promotes neonatal immune tolerance in response to antigenic stimulation by increasing the proportion of Tregs while reducing the proliferation of T helper cells and cytokine production (177). This may be one mechanism of considerable importance to ensure lifelong health.

### Human Milk Oligosaccharides (HMOs) in Breast Milk

Human milk contains substantial amounts of HMOs, which are fucosylated or sialylated. These complex carbohydrates are one of the most important biologically active compounds that are present in human milk playing essential roles in the development of the newborn (178, 179). They act as prebiotics in the establishment of the infant gut microbiota and inhibit for example the expansion of *B. Streptococci*, which can cause invasive infections in neonates (180, 181). HMOs also protect against NEC, a widespread and serious gastrointestinal condition affecting predominantly premature infants, leading to the destruction of the intestinal barrier (182, 183). Interestingly, the level of HMOs in breast milk was recently shown to negatively correlate with the incidence of this disease (184, 185). HMOs increase the intestinal mucin level thereby reducing bacterial attachment to the gut epithelium and the risk to develop NEC (186). Remarkably, not only the incidence of pathogen-mediated illnesses is influenced by HMOs, but also the onset of immune-mediated etiologies, such as autoimmune type 1 diabetes (187). Overall, the protective capacity of HMOs raises questions such as: Could additional HMO supplementation during early life be used as a therapeutic strategy for the treatment or prevention of diseases?

HMOs are known to support the development of neonatal immunity. They positively affect the expansion of intestinal commensals, which is part of our first line defense strategy by providing colonization resistance and contributing to mucosal homeostasis (179, 188). Furthermore, HMOs directly support the intestinal barrier function by affecting the maturation of epithelial cells (179). *In vitro* studies revealed that they protect the gut barrier in a dose-dependent manner by conferring resistance against inflammation-induced epithelial cell dysfunction (189).

The contribution of HMOs to immune cell development and function is also well established (179). For instance, it was recently published that they drive the maturation of monocyte-derived dendritic cells (moDCs), which in turn stimulate the generation of Tregs. Hence, HMOs enhance immune tolerance, which may be one of the central mechanisms by which they contribute to the prevention of immune-mediated diseases in the newborn (187, 190).

Apart from the immune-related and prebiotic functions of HMOs, which have long been recognized and accepted, they are also crucial for the infant’s nutrition. Recent publications display associations between the breast milk HMO composition and infant growth (191). Moreover, its supplementation during childhood has been proposed to be a promising tool to support the development and even improve growth in undernourished infants (192, 193). Additionally, HMOs were suggested to have beneficial effects on brain development by altering the expression of several genes relevant to improve recognition memory (194).

To sum up, HMOs are fundamental breast milk components for the newborn when it comes to the development of its first line defense mechanism by promoting intestinal barrier function and contributing to immune cell maturation, both key factors for guaranteeing lifelong health.

### Proteins and Peptides in Breast Milk

A broad range of peptides and proteins is found in human milk, which are involved in nutrient absorption (amylase, α1-antitrypsin), have immune and antimicrobial properties (immunoglobulins, lactoferrin, cytokines), and possess the ability to promote growth (e.g. epidermal growth factor (EGF)). Among all, α-lactalbumin, casein, lysozyme, and sIgA are the most abundant ones (167, 195, 196). As previously discussed, the neonatal immune system is inexperienced and immature. Thus, neonates rely on passive immunization through breast milk by maternally derived antibodies that offer effective and specific protection against pathogens (171). Lately, it has been demonstrated that maternal IgA prevents NEC in preterm infants by binding to intestinal bacteria (172). Hence, breast milk additionally sustains host-microbiota homeostasis, which is key for the establishment and maintenance of an equilibrated immune system. However, the binding of maternal antibodies to bacteria in the neonatal intestine not only prevents infections but also diminishes immune responses towards commensals by limiting T cell-mediated reactions early in life. An *in vivo* study found that commensal specific antibodies are transferred from the mother to the offspring *via* breast milk and persist in the offspring until weaning. In addition, mice born to antibody-deficient mothers had higher numbers of activated follicular T helper cells, which was accompanied by an increase in germinal center B cells in MLNs and Peyer’s Patches. This demonstrated that maternal commensal-specific antibodies delivered to the newborn *via* breast milk dampen host-mediated commensal specific T cell responses in the offspring, thereby contributing to mucosal homeostasis (197).

Generally, milk-derived proteins are considered essential contributors to the first line defense strategy. For instance, lactoferrin, which is part of the innate immune system, has important antimicrobial and immunomodulatory properties that support health and prevent disorders in the neonate (198). It elicits beneficial effects in a disease state on the intestinal barrier by stimulating the proliferation of epithelial cells and reducing the expression of pro-inflammatory cytokines in innate immune cells (199, 200). β-defensin 1, another protein in human milk, has antimicrobial functions and further promotes the differentiation of moDCs from neonatal cord-blood-derived monocytic precursors. This further drives the maturation of DCs, which thereby obtain their characteristic antigen-presenting capacity (201, 202). Moreover, a study revealed that milk fat globule epidermal growth factor VIII (MFGE8), also known as lactadherin, prevents NEC by limiting intestinal permeability and thus reinforces the barrier function (203). Accordingly, the amount of MFGE8 in breast milk correlated with the infant’s inflammatory state, with higher levels being associated with an anti-inflammatory gut environment (204).

Cytokines are essential contributors to the immune response, which stimulate the differentiation and maturation of various immune cells. The cytokine TGF-β is found in breast milk and is ingested by the neonate, where it stimulates mucosal IgA production and inhibits the synthesis of pro-inflammatory cytokines (205). TGF-β supplementation by oral gavage during the suckling period promoted immune maturation. It lowered NK cell frequency in the MLNs and altered the cytokine profile in the neonate (206). The same group showed that oral administration of TGF-β during this period of life further modified the splenic lymphocyte composition, suggesting effects on systemic immunity (207). Furthermore, breast milk levels of TGF-β negatively correlated with the occurrence of eczema in neonates (208). This supports the assumed potential of maternally derived cytokines to drive the maturation of the neonatal systemic and mucosal immune system. IL-7 in breast milk correlated with thymic development in the offspring (209).

Last, milk contains EGF, which was shown to prevent the weaning reaction (19). This finding suggests that breast milk may be involved in determining the duration of the previously discussed window of opportunity. Additionally, milk-derived EGF has been attributed protective features by inhibiting the formation of GAPs during the early postnatal phase, which in turn prevents the translocation of gut bacteria and thus, systemic pathogen dissemination (210). Nevertheless, and as previously mentioned, the formation of GAPs must also happen during a precise time window before weaning to develop lifelong tolerance to the gut bacteria (142). This switch to inhibit GAP formation around weaning may be fine-tuned by breast milk since the levels of milk EGF decrease throughout lactation. Overall, breast milk may not only protect the neonate but also timely regulate the different immune developmental steps (142, 210).

### Exosomes/miRNAs in Breast Milk

Exosomes are endosome-derived extracellular vesicles that are 30 - 100 nm in size and circulate in body fluids, including blood, saliva, and breast milk (211–213). They are involved in physiological and pathophysiological immune-related processes, such as antigen-presentation, immune activation, and suppression, as well as intercellular communication. Overall, they are carriers that mediate communication between different parts of the body by transferring proteins, lipids, miRNAs, and other substances (213, 214). miRNAs are small non-coding single-stranded RNA molecules, about 22 nucleotides long, which regulate gene expression and protein translation (215, 216). It is well established that they have immunoregulatory functions by interfering with inflammatory responses thereby playing a role in health and disease. Aberrant expression of miRNAs is associated with severe consequences, ranging from cell death to autoimmunity and cancer (217–220). About a decade ago, several immune-related miRNAs were discovered in breast milk and found to be highly enriched in milk-derived exosomes, suggesting that they may influence the development of the offspring (221, 222). At this time, *in vitro* studies revealed the uptake of milk-derived exosomes by human macrophages. Nevertheless, their role upon cellular absorption remains to be elucidated (223, 224). Although the capacity of cells to ingest those vesicles was demonstrated, it remained unknown whether they survive the digestive processes and whether they are eventually absorbed by intestinal cells. The latter would mean that breast milk-derived exosomes may transfer cellular components from the mother to the offspring, implying a role in the infant’s development. In more recent studies, scientists investigated whether those vesicles survive digestion by mimicking the infant’s gastric and pancreatic digestion by adjusting the pH and the addition of digestive enzymes to *in vitro* cultures. Here, milk-derived vesicles were resistant to proteolysis and survived digestion *in vitro*. They further showed that exosomal miRNAs content remained stable and were absorbed by human intestinal cells *in vitro*, suggesting the maternal-neonatal transfer of nucleic acids *via* breast milk-derived exosomes (225, 226).

The transfer of miRNA from the mother to the offspring was further investigated *in vivo*. A study conducted with pigs showed that colostrum contains higher levels of miRNA compared to mature milk and the serum of pigs, which were only fed with colostrum, had higher levels of colostrum-derived miRNAs, indicating absorption of maternally derived miRNAs (227). Another study further discovered a dose-depended increase in miRNAs in human serum post-cow milk consumption (228). In contrast, a third study did not show any evidence of miRNA absorption after milk consumption (229). These studies did not differentiate between endogenous and exogenous miRNAs. Therefore, Title et al. generated KO mice for specific miRNAs, which were then fostered by wild-type (miRNA sufficient) mothers and their results revealed no evidence for mother-to-offspring transfer of these particular miRNAs (230). In the meanwhile, a publication showed that bovine miRNAs were found in human plasma after bovine milk consumption, insinuating uptake of milk-derived miRNAs (231, 232).

A recent study using newborn calves examined the postprandial ingestion of colostrum-derived miRNAs. Colostrum, as well as maternal and calf blood, were sampled and bioavailability of colostrum-derived vesicles in calf blood and miRNA expression profiles in the different samples were assessed by small RNA-Seq. Although colostrum-derived vesicles were detected in the blood of calves, the miRNA expression profiles of the neonatal blood did not match that of colostrum. The authors consequently proposed two possible mechanisms: First, a disassembly of extracellular vesicles and a release of miRNAs, which may take place during their uptake into epithelial cells, leading to an unequal availability of vesicles and miRNAs in the circulation. Second, an imbalanced absorption of vesicle subpopulations within the colostrum. Other tissues were not analyzed for the presence of milk-derived miRNAs (233). Hence, the transfer of maternal miRNAs into the neonatal systemic circulation remains elusive and a highly discussed topic, which still needs further investigation.

The role of milk-derived exosomes and miRNAs may be of particular interest during neonatal development. The nutritional hypothesis was rapidly completed by a functional hypothesis, suggesting that they may regulate gene expression and immune processes in the newborn (234). Recently, the focus was set on investigating the ability of milk-derived exosomes to protect the mucosal epithelium during infection. Intestinal epithelial cells were incubated with H_2_O_2_, which increases oxidative stress-mediated cell death and mimics an inflammatory environment, and simultaneously treated with human breast milk-derived exosomes. The treatment had a protective effect on intestinal epithelial cells by attenuating cell death (235). Another study investigated the effect of breast milk-derived exosomes on goblet cell activity by incubating human colonic epithelial cells with bovine milk-derived exosomes. Interestingly, those vesicles promoted the expression of mucin-related genes. Moreover, they assessed whether this effect was of clinical relevance. Therefore, they induced experimental NEC in murine pups by exposing them to hypoxia and LPS and supplemented them with breast milk-derived exosomes. The treatment with milk-derived exosomes prevented intestinal injury and the reduction of goblet cells, which is a hallmark of NEC (236). Lately, human milk-derived exosomes attenuated intestinal damage and protected intestinal stem cells from undergoing apoptosis due to oxidative stress *in vitro* (237, 238). Altogether, breast milk-derived exosomes seem to protect the mucosal environment against injury and inflammation-mediated cell death by positively affecting different cell types of the intestinal epithelium.

As previously reviewed, the intestinal epithelium is protected against injury through breast milk-derived extracellular vesicles also in the context of NEC (236). An additional study could show that the administration of human breast milk-derived exosomes decreased the incidence of NEC, confirming their ability to prevent this disease (239). Furthermore, mice that were administered with milk-derived exosomes showed reduced signs of inflammation induced by DSS, which was accompanied by a lower expression of IL-6 and TNF-α, suggesting a protective effect in the context of colitis (240). Overall, existing *in vivo* studies indicate the possibility of using breast milk-derived exosomes as a potential treatment for infants with intestinal injury and NEC, or for patients with IBD.

Finally, a study investigated the direct effect on the immune system by analyzing the influence of breast milk-derived exosomes on immune cells. Human-derived peripheral blood mononuclear cells (PBMCs) were treated with milk-derived exosomes and stimulated *in vitro*. This resulted in stronger activation of NK cells as well as γδ T cells, but only in the presence of IL-2 and IL-12. This indicates that while milk exosomes alone may not activate immune cells, they may do so under inflammatory conditions (241). This could be an additional mechanism by which maternal exosomes in breast milk contribute to the prevention of immune-mediated diseases in the offspring.

### Cells Present in Breast Milk

The presence of immune cells in breast milk, including neutrophils, macrophages, and lymphocytes, is well established. Many animal studies demonstrated the transfer of those maternal cells to the neonate, and it is assumed that they contribute to the maturation of the offspring innate immune system (175, 242–244). Furthermore, flow cytometric analysis revealed the presence of ILCs in human milk, with ILC1s being the most abundant subset followed by ILC3s and ILC2s (245). While ILC1s and ILC3s are crucial to protect against bacteria and to maintain epithelial homeostasis, ILC2s play a crucial role in the defense against parasitic infections at mucosal surfaces (82, 83, 246). The role of ILCs in breast milk remains unexplored and needs further research. Another study confirmed the presence of MAIT cells and γδ T cells in human milk (247). Both cell types display features of innate immunity and are predominantly found in the gut (248), suggesting that they may influence the development of the infant’s microbiota, and thus also its first line defense strategy at mucosal surfaces.

Myeloid-derived suppressor cells (MDSCs) have repressing effects on other immune cells of the innate and adaptive immune system, such as monocytes and T cells. There are two main subsets of MDSCs, monocytic MDSCs (MO-MDSCs) and granulocytic MDSCs (GR-MDSCs) (249). Remarkably, GR-MDSCs accumulate in breast milk and suppress neonatal T cell proliferation, suggesting that milk-derived GR-MDSCs may be able to promote immune tolerance in the offspring. The same group found that GR-MDSCs reduced TLR expression on monocytes, indicating that they regulate innate immune responses in young infants (250). They also showed that the level of GR-MDSCs correlated with gestational and postnatal age, while the levels of those cells in breast milk of mothers who had delivered preterm infants were lowest (251). The authors suggested that these low levels of GR-MDSCs may contribute to reduced immune tolerance and consequently to increased susceptibility to infections, which is in line with preterm infants being at highest risk to develop NEC. Another recent study revealed that the macrophage profile in breast milk changes in response to ongoing respiratory infections in the nursing infant. The researchers observed increased frequencies of anti-inflammatory macrophages and higher IL-6 and IL-8 concentrations in the milk of mothers whose infants had an ongoing respiratory infection, indicating that the composition of breast milk changes according to the infant’s needs to ensure neonatal protection (252).

A reduction in the frequency of IL-13 producing cells in human milk has been associated with an increased incidence of atopic dermatitis in newborns (253). This cytokine is an important mediator of atopic diseases (254), implying that maternal-derived IL-13 producing cells may be involved in protecting the infant from allergy. For instance, maternal-derived IL-13 may prevent its synthesis in the offspring, thereby avoiding the activation of eosinophils and secretion of IgE, which are both central in the pathophysiological mechanism of atopy.

Apart from immune cells, breast milk contains stem cells which are more abundant in colostrum compared to mature milk (255, 256). An *in vivo* study using wild-type pups that were fostered by GFP^+^ expressing transgenic mice detected breast milk-derived stem cells in the blood as well as in the brain of the suckling pups, confirming the transfer of milk-derived cells to the offspring. Those cells differentiated into neuronal and glial cells in the pup’s brain, indicating that they play a role in the development of the offspring’s nervous system (257). Maternal immune cells found in breast milk may therefore even be implicated in the development of the infant’s immune system. However, further research is required to prove this hypothesis. A recent study using breast milk stem cells to treat spinal cord injury showed that the administration of these cells reduced apoptosis and inflammation at the site of injury, indicating their influence on immune responses and therapeutic potential (258).

### Dynamics of Breast Milk Composition

Breast milk is categorized into colostrum and mature milk, although the general components of the milk remain stable throughout lactation. As previously mentioned, the composition of milk is believed to alter, depending on the neonate’s needs. An example is that newborns are at the greatest risk to develop diseases just after birth and thus rely on maternal-derived protection. Accordingly, colostrum contains significantly higher amounts of maternally derived antibodies than mature milk. Therefore, it is widely accepted that colostrum has a principal immunologic function, while mature milk plays a more nutritional role for the neonate. Correspondingly, in humans and mice, the fat content increases throughout lactation. Hence, the milk composition adapts not only to the protective need but also to the nutritional requirements of the growing infant (195, 259).

Studies further revealed that the disease state of the mother also influences the composition of breast milk. Recently, aberrant levels of miRNAs in breast milk exosomes of diabetic mothers were found (260). Furthermore, the milk of mothers with IBD contained significantly lower levels of IgA and higher concentrations of pro-inflammatory cytokines (261). Therefore, breast milk may lead to differential immune priming of the neonate in a disease context. However, more studies are needed to investigate how breast milk affects the development of the infant’s immune system in such situations. Moreover, we have previously shown that bacteria-derived metabolites, such as AhR ligands from the maternal microbiota translocate into breast milk and contribute to the development of innate immune cells in the offspring, namely ILC3s and F4/80^+^CD11b^+^ mononuclear cells (77). Hence, the maternal intestinal microbiota can also alter breast milk composition, an area still largely elusive.

## Conclusion

In this review, we have covered the period from the very beginning of the developing embryo and fetus *in utero* up to the weaning of the young offspring and have given an overview on recent publications that show an impact of the commensal microbiota on the emerging immune system. Although we have focused on findings regarding the innate immune system, we also highlighted few key studies that demonstrated an impact on the adaptive immune system.

We hope we have convinced the reader that already during gestation the maternal microbiota can efficiently influence innate immune maturation in the developing fetus, despite the inborn character of the innate immune system. We have spread light on early life environmental factors, including birth mode and the intake of antibiotics in regard to microbiota and immune development in the neonate. We have further emphasized that the maturation of the intestinal microbiota and the evolving immune system go hand in hand and influence each other during the first few weeks (mice) or years (human) after birth. Last, we have tried to give an extensive overview on classical and non-classical immunological compounds present in breast milk and how those affect innate immune maturation in the offspring.

While evidence for an influence of the maternal microbiota, the neonate’s own microbiota or the breast milk during this critical time window is undoubtedly increasing, many aspects remain to be understood: What are the molecular mechanisms underlying the described phenotypes? How is long-term persistence into adulthood achieved? Why does the window of opportunity close around weaning, could it be reopened later in life, and how? To which extent does the maternal microbiota influence the composition of breast milk and thus immune development in the offspring? Finally, how do the maternal diet and later the offspring’s diet, and additional factors (e.g. exposure to environmental toxins or drugs) interact with the microbiota and mutually or independently influence immune maturation in the growing organism?

With our current and future research projects, we aim to complement the understanding of the window of opportunity and to use this knowledge in a preventive or therapeutic setting to improve human health from the neonatal age on.

## Author Contributions

CK, NF, SC, and SG-V wrote the manuscript and generated figures. All authors contributed to the article and approved the submitted version.

## Funding

This work was funded through a Peter Hans Hofschneider Professorship provided by the Stiftung Molekulare Biomedizin to SG-V. CK was funded through a European Research Council Starting Grant (H2020 ERC-2016-ADG HHMM_Neonates, Grant Agreement: 742195) provided to Andrew Macpherson. SC received an MD-PhD scholarship of the Swiss National Science Foundation (323530_199385).

## Conflict of Interest

The authors declare that the research was conducted in the absence of any commercial or financial relationships that could be construed as a potential conflict of interest.
